# Smaller herring larval size-at-stage in response to environmental changes is associated with ontogenic processes and stress response

**DOI:** 10.1093/conphys/coad072

**Published:** 2023-09-12

**Authors:** Léa J Joly, Maarten Boersma, Carolina Giraldo, David Mazurais, Lauriane Madec, Sophie Collet, José-Luis Zambonino-Infante, Cédric L Meunier

**Affiliations:** English Channel and North Sea Research Unit, Ifremer, 150 Quai Gambetta, 62200 Boulogne-sur-Mer, France; Shelf Sea System Ecology, Alfred-Wegener-Institut Helmholtz-Zentrum für Polar- und Meeresforschung, Biologische Anstalt Helgoland, Am Binnenhafen 1117, 27483 Helgoland, Germany; Marine Ecology, GEOMAR Helmholtz Centre for Ocean Research, Düsternbrooker Weg 20, D-24105 Kiel, Germany; Shelf Sea System Ecology, Alfred-Wegener-Institut Helmholtz-Zentrum für Polar- und Meeresforschung, Biologische Anstalt Helgoland, Am Binnenhafen 1117, 27483 Helgoland, Germany; FB2, University of Bremen, Leobener Str, 28359 Bremen, Germany; English Channel and North Sea Research Unit, Ifremer, 150 Quai Gambetta, 62200 Boulogne-sur-Mer, France; Physiology of Marine Organisms, Ifremer, Univ Brest, CNRS, IRD, LEMAR, ZI de la Pointe au Diable, 29280 Plouzané, France; Physiology of Marine Organisms, Ifremer, Univ Brest, CNRS, IRD, LEMAR, ZI de la Pointe au Diable, 29280 Plouzané, France; Physiology of Marine Organisms, Ifremer, Univ Brest, CNRS, IRD, LEMAR, ZI de la Pointe au Diable, 29280 Plouzané, France; Physiology of Marine Organisms, Ifremer, Univ Brest, CNRS, IRD, LEMAR, ZI de la Pointe au Diable, 29280 Plouzané, France; Shelf Sea System Ecology, Alfred-Wegener-Institut Helmholtz-Zentrum für Polar- und Meeresforschung, Biologische Anstalt Helgoland, Am Binnenhafen 1117, 27483 Helgoland, Germany

**Keywords:** Fish larvae, gene expression, global change

## Abstract

Global change puts coastal systems under pressure, affecting the ecology and physiology of marine organisms. In particular, fish larvae are sensitive to environmental conditions, and their fitness is an important determinant of fish stock recruitment and fluctuations. To assess the combined effects of warming, acidification and change in food quality, herring larvae were reared in a control scenario (11°C*pH 8.0) and a scenario predicted for 2100 (14°C*pH 7.6) crossed with two feeding treatments (enriched in phosphorus and docosahexaenoic acid or not). The experiment lasted from hatching to the beginning of the post-flexion stage (i.e. all fins present) corresponding to 47 days post-hatch (dph) at 14°C and 60 dph at 11°C. Length and stage development were monitored throughout the experiment and the expression of genes involved in growth, metabolic pathways and stress responses were analysed for stage 3 larvae (flexion of the notochord). Although the growth rate was unaffected by acidification and temperature changes, the development was accelerated in the 2100 scenario, where larvae reached the last developmental stage at a smaller size (−8%). We observed no mortality related to treatments and no effect of food quality on the development of herring larvae. However, gene expression analyses revealed that heat shock transcripts expression was higher in the warmer and more acidic treatment. Our findings suggest that the predicted warming and acidification environment are stressful for herring larvae, inducing a decrease in size-at-stage at a precise period of ontogeny. This could either negatively affect survival and recruitment via the extension of the predation window or positively increase the survival by reducing the larval stage duration.

## Teaser

The development and physiology of herring larvae were monitored for individuals reared in control and combined warming–acidification crossed with different food quality treatments. The experiment revealed that warming and acidification triggers a stress response at the molecular level and decrease herring larvae size-at-stage.

## Introduction

Anthropogenic activities have led to significant environmental modifications of the Earth’s system since the industrial revolution, leading to the name ‘The Anthropocene’ for our era (Lewis and Maslin, 2015). The Anthropocene is characterized by human-induced global changes, primarily from large CO_2_ emissions into the atmosphere that lead to ocean acidification (OA) (Doney *et al.,* 2009) and ocean warming (OW), which impacts marine organisms (Huey and Kearney, 2020; Barley *et al.,* 2021). In addition, indirect effects could arise, such as changes in the quality or quantity of food sources for consumers. For example, increasing temperatures reduce the proportion of essential omega-3 polyunsaturated fatty acids (n-3 PUFA) in phytoplankton (Thompson *et al.,* 1992; Guschina and Harwood, 2009; Hixson and Arts, 2016). Phytoplankton food quality is also directly influenced by dissolved nutrient availability, and dissolved phosphorus concentrations have steadily decreased in European coastal waters (Grizzetti *et al.,* 2012), resulting in an expansion of P-limited coastal zones (Sarker and Wiltshire, 2017). Thus, the quality and quantity of planktonic food have changed, and will probably continue to change, with potential cascading effects through the food chain (Boersma *et al.,* 2008; Jin *et al.,* 2020). Particularly, the predicted decrease in n-3 PUFA including docosahexaenoic acid (DHA) could threaten fish recruitment by bottom-processes (Litzow *et al.,* 2006) because DHA is involved in membrane fluidity (Niebylski and Salem, 1994), essential to brain and vision development (Tocher, 2015; Pilecky *et al.,* 2021) and metamorphosis of fish larvae (Shields *et al.,* 1999).

Because fisheries are an important part of global food production (Béné *et al.,* 2016), the potential decline in fish catch that could happen with global change (Cheung *et al.,* 2011) raises socio-economic and food security concerns. Although fish stock biomass fluctuations are affected by fishery intensity, environmental variations also play a major role because they modulate larval fish condition and associated recruitment success (Rouyer *et al.,* 2014; Polte *et al.,* 2021). The larval stage of fish is characterized by high mortality and represents a bottleneck period for many species (Houde, 2008). Hence, recruitment strongly depends on the survival and development of individuals during the larval period. Crucial morphological and physiological changes characterize the larval phase, such as organ morphogenesis and maturation of physiological functions (Zambonino-Infante *et al.,* 2008). Thus, there is a strong need to understand how global change, especially the interaction between warming and acidification on the one hand, and prey quality on the other, affect larval fish survival and development.

North Sea Atlantic herring (*Clupea harengus*) is an ecologically and socio-economically important fish species, with highly variable recruitment success (Payne *et al.,* 2013). Assessing how the predicted co-exposure of warming, acidification and change in food quality could affect development and metabolism early in life, and subsequently the sustainability of fish population, is of crucial interest, particularly to implement conservation and management tools. The first aim of the present study was to investigate the influence of global change drivers (temperature, pH, food quality) on growth in terms of changes of total length in function of time (i.e. size-at-age) and development in terms of morphological changes, specifically notochord flexion and fin development (i.e. size-at-stage) of herring larvae. We focused on these two criteria because temperature alone differentially increased growth rate and development rate on herring larvae (Moyano *et al.,* 2016), and combined effects of warming and acidification had no impact on these rates (Sswat *et al.,* 2018). The second aim was to assess the underlying mechanisms of herring larval response at the transcriptional level and to identify potential coping mechanisms or metabolism disruptions. Joly *et al.* (2021) have recently identified stage 3 (flexion of the notochord and development of caudal fin) as a critical period for Downs herring larvae. At this stage, the energetic reserves can be depleted (even at *ad libitum* feeding conditions) to meet the metabolic demand necessary for physiological and morphological changes. We investigated changes in lipid metabolism at the molecular level. Some species increase their energy expenditure to adjust to environmental changes (Araújo *et al.,* 2018; Wang *et al.,* 2020) resulting in a decrease in the expression of genes involved in lipid anabolism and glycogen synthesis, along with an increase in lipid catabolism. Conversely, some organisms can accumulate lipids to cope with stress (Strader *et al.,* 2020) or because of a disruption of the lipid metabolism leading to abnormal lipid accumulation, as found in several acidification and pollution experiments for cod and medaka larvae (Frommel *et al.,* 2012, 2014; Sun *et al.,* 2019). Finally, heat shock proteins (HSPs) expression were assessed to determine if the treatments were sources of stress for the larvae. Under stress, HSPs are synthesized and function as molecular chaperones, involved in the maintenance of protein homeostasis (Wickner *et al.,* 1999); they prevent protein aggregation and apoptosis (Roberts *et al.,* 2010). In this study, we investigated changes in gene expression related to metabolism (aerobic, lipid and glycogen) and stress response, with expectations of differential regulation of genes involved in metabolism between treatments and higher level of HSPs transcripts in the 2100 scenarios.

## Materials and Methods

### Strip-spawning and eggs incubation

North Sea herring comprises different spawning components (Geffen, 2009); here, we focused on the Downs winter component. Mature wild Downs herring were collected from local fishermen (Coopérative Maritime Etaploise, Boulogne-Sur-Mer, France, 22 November 2019). Mature females (n = 324, mean length: 25.8 ± 2 (SD) cm; mean weight: 163.8 ± 44.7 g) and males (n = 221, mean length: 25.7 ± 2 cm; mean weight: 163.5 ± 43.4 g) were strip-spawned on PVC plates. The eggs and the sperm of at least 10 females and 5 males were mixed on one PVC plate and incubated for 10 minutes in natural filtered seawater (11°C, natural pH). A total of 30 PVC plates were prepared and incubated for 48 hours in 70-l tanks (13°C, natural pH). The eggs were transferred by car to the Ifremer Centre de Bretagne Laboratory (agreement number: B29–212-05) and incubated in 200-l tanks (10.6°C, natural pH). The natural seawater for the experiment was pumped from the Bay of Brest, France (20-m depth, 32.5 salinity, pH 8.0, pCO_2_ ~500 μatm in winter; Bozec *et al.,* 2011), filtered through sand, temperated, degassed, filtered through a membrane (mesh: 2 μm) and finally UV-sterilized (PZ50, 75 W, Ocene, France). After 11 days of incubation, 21 000 larvae were randomly distributed over twelve 38-l flow-through tanks with a water flow of 20 L·h^−1^. The experiment followed the French national regulations and was authorized by the regional ethics committee (authorization number: 22555–2 019 102 319 251 011).

### Experimental design

The 12 experimental tanks were divided into four treatments with three replicates each. Because global change simultaneously affects temperature and pH, we chose to combine OA and OW. The control treatment had a constant temperature set at 11°C associated with natural seawater pH of 8.0. The second treatment (ocean warming and acidification [OWA]) corresponded to the SSP5–8.5 (IPCC, 2021) scenario (Δ + 3°C, Δ − 0.4 pH units), thus yielding a temperature of 14°C and a pH of 7.6. The seawater temperature was increased from 11 to 14°C through 48 hours, and each tank was supplied with water by a header tank (200 l) where CO_2_ was bubbled through the water column via a gas diffuser. CO_2_ diffusion was manually controlled by a flow meter and bubble counter to adjust the pH at 7.6. Temperature and pH were manually measured and adjusted two to three times a day (WTW 330i, Xylem Analytics Germany). Total alkalinity (TA) was measured once a week according to the protocol of Anderson and Robinson (1946) and Strickland and Parsons (1972). The excel macro CO2sys (Lewis and Wallace, 1998) was used to calculate pCO_2_ from water chemistry (Mehrbach *et al.* (1973) refit by Dickson *et al.* (2007) constant). Oxygen saturation (WTW Oxi 340, Xylem Analytics Germany) and salinity (WTW LF325, Xylem Analytics Germany) were measured once a week (Supplementary Table 1). Oxygen saturation was >88% during the whole experiment. The larvae were reared under a 10:14-hour light:dark cycle, with the light dimmed at dusk and dawn.

To test the consequences of change in food quality expected for the future in the North Sea (Sardans *et al.,* 2012; Boersma *et al.,* 2015), the environmental treatments (control vs OWA) were crossed with two different integrated feeding treatments. Because temperature is a driver of metabolic rate, the feeding sequences duration for each type of prey and the time of sampling were chosen using the cumulative degree days (CDD) approach (Chezik *et al.,* 2014). CDD is the cumulative sum of average temperatures encountered per day by the larvae since hatching (0 CDD). Here, 0 CDD corresponds to the hatching and 35 CDD is the mean sampling point before the distribution of larvae in the different conditions. Larvae were fed *ad libitum* following the feeding sequence used by Joly and co-authors Joly *et al.* (2021) for Downs herring with a succession of *Rhodomonas salina*, freshly hatched Artemia nauplii and 24-hour-old enriched Artemia. *Rhodomonas salina* (Strain 2002, University of Göttingen, Germany) were grown under constant light in enriched Conway*2 medium (double quantities of N and P). For the nutrient-poor treatment (*R. salina* P−), cells grown in the previous medium were isolated and grown 48 hours in a modified enriched Conway*2 medium without phosphorus. The phytoplankton was supplied between 60 and 146 CDD. Freshly hatched Artemia nauplii A0 (VNBS, Viepearl) were supplied to the larvae between 110 and 303 CDD. Artemia nauplii A1 were either enriched 24 hours with a diet (Larviva Multigrain, Biomar) containing a high content of DHA, a polyunsaturated fatty acid (22:6n-3, DHA; +2%) for the PUFA+ treatment, or in a mixture of fish oil and baker yeast for the PUFA treatment. A1 Artemia were supplied from 259 CDD. Larvae were reared from hatching to the beginning of stage 4 (post-flexion stage), corresponding to 47 dph at 14°C and 60 dph at 11°C (~663 CDD).

### Sampling

**Figure 1:**
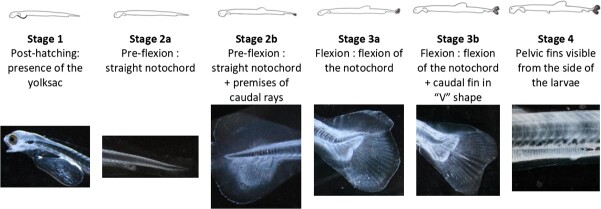
Illustration of herring developmental stages and sub-stages adapted from Doyle (1977).

Total length (millimetres) and developmental stages were measured and observed at five points during the experiment. The developmental stage characterizations were based on Doyle (1977) and modified for rapid identification under a binocular (Fig. 1).

The stage 1 identification criteria are the presence of the yolk sac (endogenous reserve). Stage 2 starts when all the yolk sac is resorbed, the notochord is straight and the distinction between stage 2a and 2b is the presence of caudal rays for stage 2b. Stage 3 includes the notochord flexion process, stage 3a includes all the stage of the flexion, and stage 3b is the end of the flexion associated with a ‘V’ shape of the caudal fin. The last developmental stage reached during this experiment is the beginning of stage 4—the pelvic fins are visible but not fully developed yet.

Larvae were sampled at five sampling points, to sample key developmental stages, and euthanized in ice water. At sampling T_0_, 35 stage 1 larvae were collected between 2 and 3 dph to obtain the mean size before tank transferring. Sampling T_1_ at 89 CDD targeted stage 1 and 2A (~20 larvae by tank), T_2_ at 253 CDD targeted stage 2B (~60 larvae by tank), T_3_ at 436 CDD stage 3A (~20 larvae by tank) and finally T_4_ at 663 CDD targeted stages 3B and 4 (~90 larvae by tank). The objective was to describe the increase in size as a function of time (dph) not only to characterize overall growth rates but also to characterize the physiological growth in relation to ontogeny and developmental stages (as a function of CDD). Developmental stage and size were described for a total of 2351 larvae throughout the experiment. The exact number of larvae sampled by treatment and sampling point, along with the correspondence between sampling points in dph and in CDD can be found in Supplementary Table 2.

### Gene expression analysis

For molecular analysis, seven larvae per tank were sampled at T_3_, stored in RNA later and kept at −20°C. Among them, only stage 3A and 3B larvae were used to focus on stage 3. Between 2 and 3 individuals were pooled by tank in function of their sub-stages to obtain a minimum wet weight of 30 mg to facilitate RNA extraction (number of pool by treatments: control = 5, control+ = 5, OWA = 2, OWA+ = 3). Total RNA was extracted from pooled samples using NucleoSpin RNA® kit (Macherey-Nagel, Germany). RNA concentration and purity were assessed by spectrophotometry using NanoDrop 2000 (ThermoScientific, USA) and RNA quality with Bioanalyseur 2100 (Agilent Technologie Inc, USA). Relative levels of mRNA expressions were quantified by reverse transcription polymerase chain reaction (RT-qPCR) analysis. The RNA extracted were reverse-transcribed in cDNA using iScript® cDNA Synthesis kit (Bio-Rad Laboratories, USA). QPCR was used to quantify the relative levels of 16 transcripts involved in different targeted metabolic pathways; selecting genes for cDNA sequences were available in the National Center for Biotechnology Information (NCBI) database (Table 1). The specific primers for each gene were designed with Primer 3 plus software (https://www.bioinformatics.nl/cgi-bin/primer3plus/primer3plus.cgi) from sequences in the NCBI database. Each biological pooled sample was analysed in technical triplicates using iQ SYBR® Green Supermix (Bio-Rad Laboratories, USA). The relative quantities of transcripts in larvae were normalized with the ΔΔCt method using the CFX-manager software associated to the CFX96 Touch Real-Time PCR Detection system (Bio-Rad Laboratories Inc.). Initially, EF1a, Rpl13 and Actin were selected as control genes for normalization of gene expression, but only Rpl13 and Actin expression were used as reference genes because their expression profiles were stable among samples (coefficient of variation and expression stability M values <25% and 0.5, respectively.).

**Table 1:** Presentation of the selected genes for investigation, the biological process they are associated with and primer specification for real-time qPCR

**Abbreviation**	**Full name**	**Function/biological process**	**Reference (NCBI database)**	**Primer sequence 5′-3′**
**EF1a**	Elongation factor 1-alpha	Elongation factor Protein biosynthesis	XM_012840422.2	F: AGTACCCTCCACTGGGTCG R: GTGGAGTTGGGTGACCTCTG
**CS**	Citrate synthase	Krebs cycle	XM_012824897.2	F: *TTGCGCCGAAGATCCTGAAT* R: *TGCCACCATACACCATGTCG*
**Idh1**	Isocitrate dehydrogenase 1	Krebs cycle	XM_012819824.2	F: TCCACTAACCCCATTGCCTC R: CCCCTTGATACAAGCTGCCA
**Idh2**	Isocitrate dehydrogenase 2	Krebs cycle	XM_012833616.2	F: CACTGTCTTCCGTGAGCCAA R: CTGTTGCTTTGTACTGGTCGC
**Dgat2**	Diacylglycerol O-acyltransferase 2	Triacylglycerol biosynthesis	XM_012827712	F: TACTTCCCCATCCGGCTCAT R: ATCCGGAAATTCCCAGCCAG
**Fasn**	Fatty acid synthase	Fatty acid biosynthesis	XM_012814622.2	F: ATCATCACTGGTGGCCTTGG R: TAGCTTGGTATCCGTTGCGG
**Lipe**	Hormone-sensitive lipase	Triacylglycerol degradation	XM_012839365.2	F: CACCAGTCTGGCATAGGAGT R: AGCTCAGGGTCTATGGCGTA
**Pnpla2**	Patatin-like phospholipase domain containing 2	Triacylglycerol degradation	XM_012840949.2	F: TCTGATCCAGTCGCTAGGCA R: CCACATGGTACGAGAAACGTG
**Gys2**	Glycogen synthase 2	Glycogen biosynthesis	XM_012824488.2	F: CTGCACAGGAACCCAGATGT R: GGACAAACTCCTGGATGCGA
**Igf1-x1**	Insulin-like growth factor 1, transcript variant x1	Growth factor	XM_012830244.2	F: CCTGCGCAATGGAACAAAGT R: GACAGCACATGGTACACTTGA
**IgfII-x1**	Insulin-like growth factor II, transcript variant x1	Growth factor	XM_031564361.1	F: GCTGAAATCAGAGTGATGTCCT R: GCCGGTCGGTCTACTGAAG
**IgfII-x2**	Insulin-like growth factor II, transcript variant x2	Growth factor	XM_012832374.2	F: CGCAGCACAAACAAGGCTAC R: TGCCGGTCGGTCTACTGAAG
**SerpinH1-like1**	Heat Shock Protein 47 - Serpin1a	Chaperone Stress response	XM_012832341.2	F: *AGCATAGTGCGGTGAACTCC* R: *GAAAGCCATAGATGCCAGTGC*
**SerpinH1-x1**	Heat Shock Protein 47 - Serpin1b	Chaperone Stress response	XM_031573303.1	F: *ATTGTTCTCGAGACATCCGC* R: *TTGCCACGTTGTGGTACAGG*
**Rpl13**	Ribosomal protein L13	Housekeeping gene	XM_012817263.2	F: CATGGCCCCCAGTAGGAATG R: CGAGCCTTATGTCTGCGTTG
**Actin**	Actin cytoplasmic 1	Housekeeping gene	XM_012839274.2	F: TCAGCGCTCCTAATCCCAAA R: CCACCATCACACCCTGATGTC

### Statistical analysis

Due to a mistake in the feeding treatment for one tank in the OWA treatment, all the larvae from this tank were excluded from the study.

Linear mixed effect models (LMEM) were used to estimate the effect of the different scenarios on the growth rate (changes in size as a function of dph including T_0_, equation 1), the physiological growth (changes in size as a function of CDD, equation 2 and 3) and the level of gene expression (equation 4). For all models, abiotic drivers (‘Enviro’) and food treatments (‘Food’) were used as fixed factor, with two levels corresponding to the crossed treatments. The models were run over all the data and the tank-replication level was included as a random effect.

Effect on total length over time in dph:

(1) TL ~ dph + Enviro + Food + Enviro:Food + CDD:Enviro + *(*1*|tank)*

The value of the slope and fixed effects were used to determine growth rates.

Effect on total length over time in CDD:

(2) TL ~ CDD + Enviro + Food + Enviro:Food + CDD:Enviro + *(*CDD*|tank)*

Effect on total length at each sampling date:

(3) TL_M_DD_ ~ Enviro + Food + Enviro:Food + *(1|tank)*

Effect on gene-relative differential expressions:

(4) Gene(X) ~ Enviro + Food + Enviro:Food + *(1|tank) + (1|stage)*

Homoscedasticity of the residuals was graphically verified for all models. Statistical analyses were performed in R using the package ‘*lmerTest*’ (lmer, Kuznetsova *et al.,* 2017). The data are expressed as ± standard deviation. The entire results are written in Supplementary Table 3, 4, 5 and 6.

## Results

### Growth, development and mortality event

**Figure 2:**
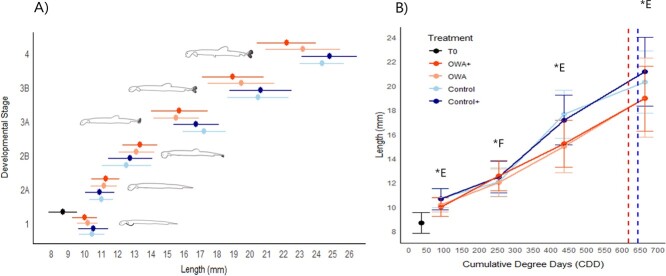
(A) Size-at-stage of herring larvae reared in four experimental treatments. The points represent the mean size and the line the range of size of the larvae from the minimum to the maximum. (B) Size variation of herring larvae sampled at five sampling points in function of CDD, in four experimental treatments. The error bars represented the standard deviation. In blue are represented the larvae reared at 11°C and pH 8.0 (control/control+), in orange the ones reared at 14°C and pH 7.6 (OWA/OWA+). The “+” represents an enriched diet in phosphorus and DHA. The (*) marks a significant difference due to “E” Environment treatment and “F” Food treatment. The red dashed lines indicate a mortality event in OWA and OWA+. The blue dashed lines indicate a mortality event in control and control+.

A mortality event was observed close to the end of the experiment, in all the tanks, at 44 dph for the OWA and OWA+ treatments and at 57 dph for the control and control+. These events occurred roughly at the same physiological–temperature-corrected time (between 610 and 630 CDD; dashed lines in Fig. 2b), with 201, 236, 139 and 254 dead individuals for OWA, OWA+, control and control+, respectively. For all treatments, a sub-sample of these dead larvae (n ~ 22–60) was used to characterize their developmental stage. Between 90 and 100% of the larvae were in stage 3B for all treatments, with a mean size of 18.07 ± 1.99 mm for OWA+, 17.33 ± 2.31 mm for OWA, 18.69 ± 2.40 mm for control and 17.86 ± 2.27 mm for control+.

Growth rates estimated by the LMEM were of 0.23 mm·d^−1^ in OWA and OWA+ treatments (LMEM, dph, χ^2^ = 7878.15, *df* = 1, *P* < 0.001) and 0.21 mm·d^−1^ in both control and control+ treatments (LMEM, dph:Enviro, χ^2^ = 6.11, *df* = 1, *P* = 0.013).

Two main patterns were observed in the size-at-stage of herring larvae during the experiment (Fig. 2a and Table 2). Figure 2a summarizes the size–class distribution for each larval stage, all sampling combined, and Table 2 describes precisely the proportion of larva and length information at each developmental stage for each sampling point. For stages 2A and 2B, the mean size-at-stage was close between all treatments, with only slightly bigger larvae in the OWA treatments (mean length, 2A: 11.14 ± 0.79 mm and 11.25 ± 0.86 mm; 2B: 13.12 ± 1.1 mm and 13.34 ± 1.0 mm for OWA and OWA+, respectively) when compared with the control scenarios (mean length, 2A: 11.00 ± 0.72 mm and 10.89 ± 0.88 mm; 2B: 12.5 3 ± 1.5 mm and 12.75 ± 1.35 mm for control and control+, respectively). Conversely, for stages 3A, 3B and 4, mean size-at-stage was always smaller in the OWA scenarios (mean length, 3A: 15.52 ± 1.37 mm and 15.72 ± 1.70 mm; 3B: 19.44 ± 2.00 mm and 18.94 ± 1.87 mm, stage 4: 23.19 ± 2.26 mm and 22.19 ± 1.78 mm, for OWA and OWA+, respectively) than in control scenarios (mean length, 3A: 17.22 ± 1.31 mm and 16.73 ± 1.36 mm; 3B: 20.48 ± 1.85 mm and 20.63 ± 1.88 mm, stage 4: 24.33 ± 1.34 mm and 24.79 ± 1.68 mm, for control and control+, respectively). Larvae reached the post-flexion stage (stage 4) at 22 mm in control and 21 mm in control+ against 19 mm for OWA and OWA+ (Table 2).

**Table 2:** Length (millimetres) for herring larvae reared in four treatments and sampled at five points during the experiment (T_0 − 5_). Larvae were reared at 11°C and pH 8.0 (control/control+) and at 14°C and pH 7.6 (OWA/OWA+). The proportion of each stage is indicated for each sampling point

**(Sample) CDD**	**Treatment**	**Stage**	**n**	**Proportion**	**Length (mm)** **Minimum**	**Length (mm)** **Maximum**	**Length (mm) Mean**	**Length (mm)** **SD**
**(T**_**0**_ **) 35**		1	35	100.0%	7	10	8.69	0.87
**(T**_**1**_ **) 89**	**Control**	1	42	61.8%	8	11	10.43	0.77
2A	26	38.2%	10	12	11.15	0.46		
**Control+**	1	37	54.4%	8	12	10.51	0.9	
2A	31	45.6%	7	12	10.81	0.87		
**OWA**	1	42	95.5%	9	11	10.17	0.62	
2A	2	4.5%	10	11	10.5	0.71		
**OWA+**	1	67	100.0%	8	11	9.99	0.75	
**(T**_**2**_ **) 253**	**Control**	2A	21	10.6%	9	12	10.81	0.93
2B	177	89.4%	8	15	12.2	1.12		
**Control+**	2A	17	8.6%	9	13	11.06	0.9	
2B	179	90.4%	9	16	12.58	1.25		
3A	2	1.0%	15	15	15	*NA*		
**OWA**	2A	67	50.8%	9	13	11.16	0.79	
2B	65	49.2%	11	14	12.88	0.74		
**OWA+**	2A	63	31.8%	10	13	11.25	0.86	
2B	135	68.2%	10	15	13.17	0.85		
**(T**_**3**_ **) 436**	**Control**	2B	16	20.5%	12	17	15.25	1.48
3A	45	57.7%	15	20	17.69	1.18		
3B	17	21.8%	18	22	19.82	1.42		
**Control+**	2B	13	16.7%	13	16	14.38	1.19	
3A	44	56.4%	12	19	17.02	1.42		
3B	21	26.9%	17	21	19.19	1.29		
**OWA**	2A	1	1.9%	11	11	11	*NA*	
2B	14	26.9%	10	15	13.14	1.7		
3A	26	50.0%	13	17	15.08	1.16		
3B	11	21.2%	14	21	17.55	1.75		
**OWA+**	2B	22	28.2%	10	16	13.45	1.41	
3A	39	50.0%	13	19	15.21	1.26		
3B	17	21.8%	16	20	17.59	1.18		
**(T**_**4**_ **) 663**	**Control**	2B	6	2.1%	14	16	15	0.89
3A	31	10.7%	14	19	16.55	1.21		
3B	228	78.9%	13	25	20.53	1.87		
4	24	8.3%	22	27	24.33	1.34		
**Control+**	2B	5	1.7%	13	16	14.6	1.14	
3A	18	6.2%	15	18	16.22	0.94		
		3B	209	72.6%	15	25	20.78	1.87
		4	56	19.4%	21	30	24.79	1.68
	**OWA**	2B	17	8.9%	12	16	14.06	1.03
	3A	37	19.3%	13	19	15.84	1.42	
	3B	106	55.2%	15	25	19.64	1.93	
	4	32	16.7%	19	27	23.19	2.26	
	**OWA+**	2B	13	4.5%	13	16	14.85	1.14
	3A	46	16.0%	12	21	16.15	1.92	
	3B	181	62.8%	15	24	19.08	1.88	
	4	48	16.7%	19	26	22.19	1.78	

LMEM indicate that abiotic factors (T° and pH) and CDD have a significant effect on the size of herring larvae (LMEM, CDD*Enviro, χ^2^ = 12.36, *df* = 1, *P* < 0.001). The food treatment induced a small difference only at T_2_ (LMEM, Food, χ^2^ = 5.31, *df* = 1, *P* = 0.021). Differences in size due to the environmental treatments were significant at T_1_ (LMEM, Enviro, χ^2^ = 21.91, *df* = 1, *P* < 0.001), T_3_ (LMEM, Enviro, χ^2^ = 33.27, *df* = 1, *P* < 0.001) and T_4_ (LMEM, Enviro, χ^2^ = 9.04, *df* = 1, *P* < 0.01), they were more important for the last two samplings. At T_3_, the mean size in control+/control was 17.41 ± 1.59 mm against 15.14 ± 1.54 mm in OWA+/OWA, regardless of the development stage; thus, a difference in size of 13%. Larvae were mostly in stage 3A (≥50%, Table 4) at T_3_, and the difference in size was similarly 12.7% for this specific stage. The size difference was 8% at stage 4 between control+/control and OWA+/OWA at the end of the experiment (Table 2).

The change in larval size between treatments happened specifically between stages 2B and 3A (Fig. 2a) and specifically between 253 and 436 CDD (Fig. 2b).

### Gene expression profiles

The relative expression of genes involved in key mechanisms was measured by qPCR for larvae in stage 3 (3A and 3B) at 436 CDD.

**Figure 3:**
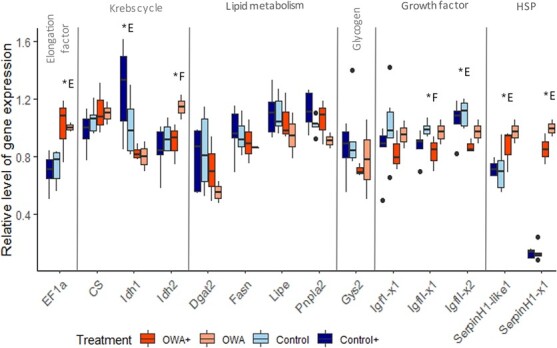
Gene expression profiles in four experimental treatments for stage 3 herring larvae. In blue are represented the larvae reared at 11°C and pH 8.0 (control–control+), in orange the ones reared at 14°C and pH 7.6 (OWA-OWA+). The “+” represents an enriched diet phosphorus and DHA. The (*) marks a significant difference due to “E” Environment treatment and “F” Food treatment.

LMEM revealed that the expression of 7 genes (of 14) was differentially regulated between treatments. The regulation was significant but low (Fig. 3) for EF1a (LMEM, Enviro, χ^2^ = 6.29, *df* = 1, *P* = 0.012), Idh1 (LMEM, Enviro, χ^2^ = 8.76, *df* = 1, *P* = 0.003), Idh2 (LMEM, Enviro, χ^2^ = 1.65, *df* = 1, *P* = 0.003; Food, χ^2^ = 4.76, *df* = 1, *P* = 0.03), IgfII-x1 (LMEM, Food, χ^2^ = 6.72, *df* = 1, *P* = 0.009), IgfII-x2 (LMEM, Enviro, χ^2^ = 5.90, *df* = 1, *P* = 0.015) and SerpinH1-like1 (LMEM, Enviro, χ^2^ = 7.76, *df* = 1, *P* = 0.005),with a difference inferior at 0.5 relative level of expression. The elongation factor EF1a, initially selected to be a reference gene, had a higher expression in OWA and OWA+ treatments (Fig. 3). Among the genes playing a role in the Krebs cycle, idh1 was higher in the control environment and idh2 in scenarios with the non-enriched diet. Within lipid and glycogen metabolism, none of the expression of the genes tested was modified. The expression of growth factors IgfII-x1 and IgfII-x2 was higher in the non-enriched diet treatments and higher in control environment, respectively. The expression of SerpinH1-like1 was higher in OWA environment. The main difference was for the SerpinH1-x1 (heat shock protein 47, serpin1b), which displayed almost a 7-fold increase in the OWA environment in comparison with the control one (LMEM, Enviro, χ^2^ = 457.1, *df* = 1, *P* < 0.001).

## Discussion

In our study, we found no direct lethal effects for Downs herring larvae resulting from combined warming, acidification and food quality. However, we did observe smaller larval size-at-stage under warming and acidification scenarios (OWA, OWA+), which was associated with physiological disturbances at the molecular level through the induction of stress-response genes. The effects of abiotic environmental treatments occurred between the transition of stage 2 and 3, which was previously characterized as a potential critical period for Downs herring larvae (Joly *et al.,* 2021).

### Warming and acidification: a stressful but not lethal environment for herring larvae

The combined stressors of warming and acidification (14°C*pH 7.6), which are expected by the end of the century, were not lethal for herring larvae. This result was not unexpected, given that previous experiments have identified lethal temperatures for herring between 22 and 24°C (Blaxter, 1960). In addition, various acidification ranges tested experimentally have never been lethal for herring larvae (Frommel *et al.,* 2014; Sswat *et al.,* 2018). The mortality event reported in our study occurred in all tanks and treatments at different times (dph) but around the same CDD, which suggest that external factors, such as water pollution, are unlikely to be the cause. The mortality was relatively homogenous between the treatments and occurred toward the end of stage 3, where herring larvae switch from a larval to an adult mode of digestion (Joly *et al.,* 2021). It rather suggests a mortality phenomenon linked to ontogeny and natural processes because a disruption of the digestive tract maturation is associated with lower larval survival, lower growth and higher malformations (Rønnestad *et al.,* 2013).

Stage 3 larvae showed either low or similar expression levels for most of the genes involved in energy metabolism. Gene regulation of key enzymes involved in the Krebs cycle can reflect modifications in aerobic potential, and a decrease in some of these gene expressions could also be linked with acid–base regulatory imbalance (Pimentel *et al.,* 2020). Growth hormones expression was also investigated to be compared with larval growth rates and assess if the associated increase in growth rate with higher temperature can be observed at the transcriptional level (Madeira *et al.,* 2016). The genes involved in the Krebs cycle, lipid and glycogen metabolism were not affected by environmental stressors like warming, acidification and food quality. This suggests that the energy metabolism of herring larvae was not adjusted at the transcriptional level to cope with these stressors. However, this contrasts with previous studies on other fish larvae, which have shown transcriptional and whole-organism regulation of certain genes in response to environmental stressors (Frommel *et al.,* 2012, 2014; Sun *et al.,* 2019; Strader *et al.,* 2020). For instance, cod larvae exposed to severe acidification showed disruption of lipid metabolism and organ damage and had transcriptional regulation of genes like citrate synthase, glycogen synthase 2 and fatty acid synthase (Frommel *et al.,* 2012, 2020). In our experiment, we unexpectedly found an up-regulation of the elongation factor EF1a, which is commonly used and recommended as a reference gene for fish (Urbatzka *et al.,* 2013; Cordero *et al.,* 2016; Raposo de Magalhães *et al.,* 2021) and has already been used for Pacific herring (Incardona *et al.,* 2015, 2021). Nevertheless, previous studies have shown that EF1a can be upregulated in response to various stressors in invertebrates such as exposure to copper (Zapata *et al.,* 2009), hypoxia (David *et al.,* 2005) or low salinity conditions (Jones *et al.,* 2019). Therefore, the use of EF1a as reference gene should be used with caution in studies investigating transcriptomics regulation under warming and acidification conditions in fish larvae. The growth rates of the herring larvae were similar across all treatments, and the transcriptional expression of growth hormones was not significantly different. This suggests that larval growth was not stimulated by warming and acidification.

The major result of the transcriptomic analysis was that the heat shock protein 47 (Hsp47, serpin1b) was strongly adjusted, with higher gene expression observed in larvae exposed simultaneously to warming and acidification. The gene expression profiles provided insight into the long-term response (e.g. chronic, >4 weeks) to the OWA treatment, which represents the physiological adaptation made by the larvae after the initial stress response, with potential implications for fitness (Oomen and Hutchings, 2017). Hsp47 belongs to the family of low molecular weight HSPs (Basu *et al.,* 2002), primarily induced during stress (Ciocca *et al.,* 1993). HSPs are not only activated for heat tolerance and thermal acclimation (Mahanty *et al.,* 2017) but also provide protection against other stressors, such as acidification (Mittermayer *et al.,* 2019) or pollutants (Mitra *et al.,* 2018). Increase in hsp47 transcript played a key role in the survival of a minnow species exposed to a warmer environment for a long time (Mahanty *et al.,* 2017). The synthesis of HSPs is essential for coping with stress, but it comes at an energetic cost (Harianto *et al.,* 2018) that may affect the development and survival of a species, leading to potential trade-offs (Somero, 2012).

### Reaching stage 4 at smaller size: phenotypic plasticity or trade-off in energy allocation?

Growth and developmental rates are temperature-dependent traits, reflecting molecular thermodynamics and rate of biochemical reactions (Havird *et al.,* 2020). The effect of temperature on body size and larval phase duration is a well-known process for herring (Johnston *et al.,* 2001; Moyano *et al.,* 2016). Development generally has a stronger temperature dependence (Forster *et al.,* 2011; Kamiński *et al.,* 2013), reducing the larval phase duration, which can lead to a smaller size at the same developmental age (Green and Fisher, 2004), as observed in our experiment. Here, warming and acidification did not strongly impact growth rate but induced a developmental acceleration as larvae reached the post-flexion stage at 47 dph at 14°C*pH 7.6 against 60 dph at 11°C*pH 8.0, resulting in smaller larvae. The response of these traits for herring larvae seems to be study-dependent and are mostly reported for single-stressor experiments. On one hand, warming increased larval growth rate (millimetre per day) for Baltic herring but still reduced the larval size-at-stage (Moyano *et al.,* 2016), suggesting than the effects on the developmental rate were higher than effects on growth rate. On the other hand, acidification alone also reduced size-at-stage in Atlantic herring and significantly reduced growth (Frommel *et al.,* 2014). In another case, Sswat and co-authors (Sswat *et al.,* 2018) showed no effect of combined warming and acidification on growth and development rate of Atlantic herring. In our study, the difference in size-at-stage, for stages 3A, 3B and 4, was not due to a reduction in growth rate *per se* but to a decoupling between growth rate and development rate in the stressful environment.

A stable growth rate (0.21–0.22 mm·d^−1^), between 11 and 14°C suggests a strong acclimation capacity and phenotypic plasticity of herring larvae. Downs herring larvae reared *ad libitum* and at 13°C had also a growth rate of 0.22 mm·d^−1^ (Joly *et al.,* 2021). Moreover, experimental herring larval growth rates of different stocks or sub-components are of the same order despite a larger range of temperature used. For Atlantic autumn spawners reared at 8°C, the growth rate was of 0.24 mm·d^−1^ (Johannessen *et al.,* 2000) and of 0.22 mm·d^−1^ for Clyde herring larvae reared at 9.5°C (Ehrlich *et al.,* 1976). For western Baltic spring spawners reared at 7 and 11°C, the growth rates were 0.215 and 0.224 mm·d^−1^, respectively, and only increased at rearing temperature of 15°C to reach 0.341 mm·d^−1^ (Moyano *et al.,* 2016). Most of these experimental values are also close to the one observed in the field; for western Atlantic spring spawner, the rate was 0.22 mm·d^−1^ at 6°C (Campana and Moksness, 1991). Concerning Downs herring larvae, a growth rate of 0.26 mm·d^−1^ was calculated in water between 6.7 and 10.7°C (Denis *et al.,* 2017). Our results combined with data from the literature indicate that the growth rate seems to be within the optimum thermal range for growth, either suggesting that herring larvae are eurytherms or that they are capable to compensate for the temperature effect for the range of temperature tested. The compensation could be possible because the critical thermal maximum of herring larvae has been shown to increase in warmer waters (Moyano *et al.,* 2017). This acclimation mechanism could maintain the growth rate constant at different temperatures (Havird *et al.,* 2020).

Another explanation to the lack of change in growth rates could be linked to physiological constraints and bioenergetic budget. Smaller body size in warmer aquatic environments is commonly observed (Daufresne *et al.,* 2009) and the pattern is described by the temperature–size rule (Atkinson, 1994). A decline in fish size related to warming has already been reported in the North Sea (Baudron *et al.,* 2014), but is not an universal rule (Audzijonyte *et al.,* 2020). Extensive research has investigated potential underlying mechanisms to explain the common diminution in fish size. Despite an important amount of empirical data reporting smaller size with warming in different taxonomic groups, there is currently no unifying explanation to explain the temperature and body size relationship (Clark *et al.,* 2013; Lefevre *et al.,* 2017, 2018; Jutfelt *et al.,* 2018; Audzijonyte *et al.,* 2019). The fish incapacity to meet increased metabolism energetic requirement with warming has been put forward to explain the size diminution, due to potential physiological limitation in oxygen supply (Pauly, 1981; Pörtner and Knust, 2007; Pörtner *et al.,* 2017) or limiting extrinsic factors such as food availability (Clemmesen, 1994). Although we did not measure the respiration of the larvae in the different treatments, at the transcriptional level, the general metabolism was undisturbed and the food supply was unlimited, suggesting that a potential increase in the metabolic rate and food shortage is not a strong explanation for the smaller size we observed. Herring larvae started to be smaller from stage 3A, and the shift in growth trajectory happened between 253 and 436 CDD. Interestingly, the transition between stages 2 and 3 has been identified as a critical energy-consuming period where the liver reserves are depleted even with *ad libitum* feeding (Joly *et al.,* 2021). Stage 3, in addition to being a period of intense morphological and anatomical changes (i.e. fins and gut development), corresponds to the period of enzymatic maturation of the digestive system defined between 28 and 35 dph (equivalent to 364 and 455 CDD). Overall, this period is characterized by strong ontogenic energy-demanding processes.

Because energy intake and assimilation are limited in organisms and energy acquisition, conversion or allocation can be perturbed by stressors (Sokolova *et al.,* 2012), we hypothesized that an energetic trade-off could be the main cause for the reduction in size-at-stage we observed. The coping mechanism to tolerate the combined pressure of warming and acidification by inducing cellular protection, via HSPs, likely increased the energetic demand during an already critical period of the development and reduced the amount of energy directed toward growth.

### Ecological implications for population recruitment

Linking the results of growth and transcriptomics allows us to hypothesize that herring larvae are robust and able to cope with predicted levels of global warming–acidification combined with changes in food quality. Indeed, HSPs production without further metabolism disruption indicate a strong acclimation response that can enhance survival in the face of a stress (Narum *et al.,* 2013; Mahanty *et al.,* 2017). Still, the use of this phenotypic plasticity has an energetic cost that affects growth and could be detrimental at the population level because growth is a key function to an organism’s fitness. Further research should focus on longer term experiments to investigate if reductions in size-at-stage can be compensated later in development. If this were not the case, subsequent smaller juveniles would yield smaller adults at maturity with potential negative effects for the reproductive success and fitness of individuals (Kingsolver and Huey, 2008). In addition, the condition of larvae could be investigated to detect other carryover effects that could lead to death later in the development (Pechenik, 2006). Resource availability and quality are important drivers of larval fish condition, especially when combined with abiotic stressors (Cominassi *et al.,* 2020). The *ad libitum* feeding may have mitigated the effects of the abiotic treatments we chose for this study. Moreover, although our study focused on the larval stage, it is important to consider the potential effects on the embryonic stage. Previous research has shown that incubation scenarios with varying temperatures and CO_2_ levels can impact embryonic development and influence the size and performance of hatchings (Leo *et al*., 2018). These sublethal effects observed in our study could potentially interact with the cumulative effects throughout the early life stages. Smaller sized larvae at hatching may face challenges in accessing food resources in the natural environment, which could further reduce their size at stage 3 and 4, potentially increasing mortality.

The larval phase is a period of high mortality (Hjort, 1914) mainly caused by starvation and predation (Houde, 2008). Predation pressure on larvae is size-selective, with higher rates observed on small larvae (Meekan *et al*., 2006). This observation aligns with the ‘bigger is better’ hypothesis (Houde, 2008), which argues that a higher growth rate enables individuals to reach a larger size faster and minimize their vulnerability to predation. Higher larval growth rates are often associated with increased recruitment (Jørgensen *et al*., 2014), whereas low growth rates have been linked to decreased recruitment in North Sea herring (Payne *et al.,* 2013). Regarding our results, the growth rate was slightly higher in the warming and acidification treatments, but the larval size was reduced for the same developmental stage due to an increase in developmental rate. On one hand, this could lead to increased larval mortality in the future because the predation window may be extended for late developmental stages, potentially resulting in decreased population recruitment. On the other hand, the reduction in larval phase duration could enhance survival by minimizing the time spent in this period of high mortality (Leggett and Deblois, 1994). This aligns with the ‘stage duration hypothesis’ (Houde, 2008), which focuses on the time spent in the plankton compartment and at the larval stage rather than the size of individuals. Although it is generally considered that increased growth shortens the larval phase (Shine, 1978; Jørgensen *et al*., 2014), our results highlight the need to differentiate between growth and developmental rate when considering the overall growth of organisms. In our study, the accelerated developmental rate led to increased larval mobility earlier due to fin development, which could enhance feeding success. Combined with the reduction in time spent in the pelagic environment, this may increase larval survival. To fully assess the potential effects of warming and acidification on herring in the future, it is crucial to clearly identify the current environmental drivers of recruitment.

Thus, understanding the vulnerability of fish larvae toward global change also requires considering the dynamic environment encountered during the pelagic phase and potential larval drift. From the perspective of recruitment and population sustainability, there is an urgent need to integrate physiological experimental studies and fieldwork on fish larvae development and survival to implement effective conservation tools and gain a better understanding of key mechanisms that influence larval recruitment under current and future conditions.

## Author contributions

Conceptualization: LJJ, CG, JLZI, CLM—Methodology: LJJ, JLZ, CG, CLM, MB, DM, SC—Validation: LJJ, JLZ, CG, CLM, MB**—**Formal analysis: LJJ—Investigation: LJJ, SC, LM—Resources: CG, CLM, JLZI**—**Data Curation: LJJ—Writing-original draft: LJJ—Writing, reviews and editing: MB, CLM, CG, JLZI, DM**—**Visualization: LJJ—Supervision: CG, CLM, JLZI, MB, DM**—**Project administration: CG, JLZI, CLM—Funding acquisition: CG, CLM, MB.

## Conflicts of interest

The authors declare no competing or financial interests.

## Funding

This work was supported by the project “CoCktAIL” (Climate ChAnge effects on fIsh Larvae, 2018–2022) awarded by the Alfred-Wegner-Institut; Zentrum für Marine Umweltwissenschaften; Institut Français de Recherche pour l’Exploitation de la Mer (AMI) Partnership Program and Bundesministerium für Bildung und Forschung grant [01LN1702A] to CLM and through the Bioweb project to MB.

## Data Availability

The data underlying this article will be shared on reasonable request to the corresponding author.

## Supplementary Material

Web_Material_coad072
