# Supplementary material for: Smaller herring larval size-at-stage in response to environmental changes is associated with ontogenic processes and stress response
Source: Conserv Physiol. 2023 Sep 12;11(1):coad072. doi: 10.1093/conphys/coad072 (PMC10498416; doi:10.1093/conphys/coad072)
Supplement: Web_Material_coad072 [file web_material_coad072.zip › JOLY2023SupData_final.pdf]

Supplementary data

Supplementary Table 1 – Environmental parameters (mean ± SD) of temperature (T, °C), pH, Salinity, Total Alkalinity (TA, µmol/kgSW) and pCO2 (µatm). "+" notify treatments enriched in phosphorus and docosahexaenoic acid.

Treatments	T (°C)	pH	Salinity	TA (µmol/kgSW)	pCO_2 (µatm)
Control (11°C, pH 8.0)	11.6 ± 0.4	8.03 ± 0.01	32.7 ± 0.4	2277.0 ± 117.7	562.9 ± 53.2
Control+ (11°C, pH 8.0)	11.3 ± 0.4	8.03 ± 0.05	32.7 ± 0.4	2273.3 ± 161.5	555.3 ± 59.7
OWA (14°C, pH 7.6)	14.3 ± 0.5	7.63 ± 0.11	32.7 ± 0.4	2271.8 ± 279.3	1549.3 ± 279.3
OWA+ (14°C, pH 7.6)	14.1 ± 0.4	7.59 ± 0.10	32.7 ± 0.4	2288.9 ± 185.9	1773.8 ± 264.6

Supplementary Table 2 – Time of sampling in day post hatch (dph) and equivalent mean Cumulative Degree Days (CDD). Control and Control+ environmental treatments were at 11°C and pH 8.0 (Control), OWA and OWA+ were at 14°C and pH 7.6. The "+" notify treatments enriched in phosphorus and docosahexaenoic acid.

Sampling	Treatment	dph	mean CDD	Replicates	Number of larvae sampled
**T0**	Pre-experiment	2<br>3	35	1	35
**T1**	OWA+	6	89	3	67
	OWA	6		2	44
	Control+	9		3	68
	Control	9		3	68
**T2**	OWA+	18	253	3	198
	OWA	18		2	132
	Control+	23		3	198
	Control	23		3	198
**T3**	OWA+	31	436	3	78
	OWA	31		2	52
	Control+	39		3	78
	Control	39		3	78
**T4**	OWA+	46	663	3	288
	OWA	47		2	192
	Control+	60		3	288
	Control	59		3	289

*Supplementary Table 3 – Results of a mixed-effects model testing for the effect of environmental treatment (Enviro, 11°C*pH8.0 / 14°C*pH*7.6), food quality (enriched in phosphorus and docosahexaenoic acid / not enriched), time in day post hatch (dph), and their interactions on herring larvae size.*

Aim	Response variable	Explanatory variables	χ^2	df	p-value	Fixed effect estimates	
Fish growth over time in day post hatch	TL (mm)	dph	7878.15	1	<0.001*	Intercept	8.30
		Enviro	3.58	1	0.058	dph	0.228
		Food	0.61	1	0.433	EnviroControl	-0.174
		Enviro*Food	0.01	1	0.912	Food	-0.157
		dph*Enviro	6.11	1	0.013*	EnviroControl :Food	-0.052
						Dph:EnviroControl	-0.012

*Supplementary Table 4 – Results of a mixed-effects model testing for the effect of environmental treatment (Enviro, 11°C*pH8.0 / 14°C*pH*7.6), food quality (enriched in phosphorus and docosahexaenoic acid / not enriched), time in Cumulative Degree Days (CDD), and their interactions on herring larvae size.*

Aim	Response variable	Explanatory variables	χ^2	df	p-value
Fish growth over time in cumulative degree days	TL (mm)	CDD	1645.94	1	<0.001*
		Enviro	1.47	1	0.225
		Food	1.12	1	0.291
		Enviro*Food	0.54	1	0.460
		CDD*Enviro	12.36	1	<0.001*

*Supplementary Table 5 - Results of mixed-effects models testing for the effect of environmental treatment (Enviro, 11°C*pH8.0 / 14°C*pH*7.6), food quality (enriched in phosphorus and*

docosahexaenoic acid / not enriched) and their interactions on herring larvae size at four sampling points.

Aim	Response variable	Explanatory variables	X^2	df	p-value
Size at sampling point (mean cumulative degree days)	TL_{89_DD}	Enviro	21.91	1	<0.001*
		Food	0.86	1	0.353
		Enviro:Food	0.28	1	0.594
	TL_{253_DD}	Enviro	0.02	1	0.881
		Food	5.31	1	0.021*
		Enviro:Food	0.10	1	0.751
	TL_{436_DD}	Enviro	33.27	1	<0.001*
		Food	0.18	1	0.667
		Enviro:Food	0.83	1	0.362
	TL_{663_DD}	Enviro	9.04	1	<0.01*
		Food	0.54	1	0.463
		Enviro:Food	0.58	1	0.447

*Supplementary Table 6 - Results of mixed-effects models testing for the effect of environmental treatment (Enviro, 11°C*pH8.0 / 14°C*pH*7.6), food quality (enriched in phosphorus and docosahexaenoic acid / not enriched) and their interactions on the level of gene expressions of herring larvae size at 436 cumulative degree days.*

Aim	Response variable	Explanatory variables	χ^2	df	p-value
Gene expression	EF1a	Enviro	6.29	1	0.012*
		Food	0.02	1	0.886
		Enviro:Food	0.03	1	0.853
	CS	Enviro	1.27	1	0.260
		Food	0.63	1	0.427
		Enviro:Food	1.12	1	0.289
	Idh1	Enviro	8.76	1	0.003*
		Food	1.92	1	0.166
		Enviro:Food	0.06	1	0.809
	Idh2	Enviro	1.65	1	0.003*
		Food	4.76	1	0.030*
		Enviro:Food	0.03	1	0.869
	Dgat2	Enviro	0.40	1	0.527
		Food	0.01	1	0.997
		Enviro:Food	0.10	1	0.746
	Fasn	Enviro	1.23	1	0.267

	Food	0.11	1	0.742
	Enviro:Food	0.12	1	0.730
	Enviro	1.17	1	0.278
Lipe	Food	0.18	1	0.671
	Enviro:Food	0.34	1	0.560
	Enviro	1.81	1	0.178
Pnpla2	Food	3.83	1	0.050
	Enviro:Food	0.08	1	0.774
	Enviro	1.52	1	0.218
Gys2	Food	0.64	1	0.422
	Enviro:Food	0.01	1	0.903
	Enviro	0.59	1	0.443
Igf1-x1	Food	1.60	1	0.206
	Enviro:Food	0.77	1	0.381
	Enviro	0.63	1	0.426
IgfII-x1	Food	6.72	1	0.009*
	Enviro:Food	0.01	1	0.981
	Enviro	5.90	1	0.015*
IgfII-x2	Food	1.00	1	0.317
	Enviro:Food	0.29	1	0.597
	Enviro	7.76	1	0.005*
SerpinH1-like1	Food	0.68	1	0.409
	Enviro:Food	0.42	1	0.515
	Enviro	457.10	1	<0.001*
SerpinH1-x1	Food	2.44	1	0.118
	Enviro:Food	3.23	1	0.072
